# Assessment of AlN/Mg–8Al Composites Reinforced with In Situ and/or Ex Situ AlN Particles

**DOI:** 10.3390/ma14010052

**Published:** 2020-12-24

**Authors:** Tong Gao, Zengqiang Li, Kaiqi Hu, Yihan Bian, Xiangfa Liu

**Affiliations:** Key Laboratory for Liquid–Solid Structural Evolution and Processing of Materials, Ministry of Education, Shandong University, 17923 Jingshi Road, Jinan 250061, China; tgao@sdu.edu.cn (T.G.); lee940505@126.com (Z.L.); 15169096855@163.com (K.H.); yhbian@yeah.net (Y.B.)

**Keywords:** Mg composites, AlN particles, microstructure, mechanical properties

## Abstract

In this paper, 8.2AlN/Mg–8Al composites reinforced with in situ and/or ex situ AlN particles have been synthesized. The in situ-formed AlN particles are nano-sized, performing as particle chains. It has been clarified that the in situ AlN particles are more efficient than ex situ particles for the enhancement of mechanical properties. The in situ-prepared composite exhibits improved density, hardness and compressive strength compared to the ex situ ones. This work may be referred to for designing particle-reinforced Mg composites by various methods.

## 1. Introduction

Particle-reinforced metal matrix composites (PMMCs) have attracted much attention recently, exhibiting their great potential in the automotive and structural fields [1,2,3,4,5]. Compared with Al, Mg has unique advantages acting as the matrix metal for PMMCs due to its lower density [6,7]. As widely reported, the commonly used ceramic particles to fabricate Mg matrix composites include SiC [8], TiC [9], TiB_2_ [10] etc.

In recent years, AlN-reinforced Mg composites have been continually investigated by scholars, as AlN has a low density (3.26 g/cm^3^), high elastic modulus, high thermal conductivity and good interface combination with Mg matrix [11,12]. So far, the published work on AlN/Mg composites can be summarized into two categories. One category seeks to introduce ex situ AlN through casting, pressure infiltration or powder metallurgy technologies [13,14]. The other category is trying to in situ synthesize AlN particles by introducing nitride or N_2_ gas into a Mg–Al melt. For instance, several scholars have reported AlN/Mg composites through the nitrogen gas bubbling method by utilizing the reaction between N_2_ and dissolved Al in a Mg melt [15,16,17]. In addition, in our previous work [18,19], we confirmed the feasibility of preparing AlN particle-reinforced Mg–Al alloys by using the Al–AlN master alloy as a raw material.

In this paper, three 8.2AlN/Mg–8Al composites reinforced with in situ and/or ex situ AlN particles are prepared, respectively. The strengthening efficiency of ex situ and in situ AlN particles is clarified by measuring the density, hardness, and compressive strength of the composites.

## 2. Materials and Methods

The raw materials used in this paper include commercial purity Mg powder (~75 μm; all compositions quoted in this work are in wt.% unless otherwise stated), commercial purity Al powder (~30 μm), AlN powder (~1 μm) and nitride plastid powder (~1 μm), which is mainly composed of hexagonal BN powder [20]. Firstly, the powders were weighed and mixed according to the designed content. Then, the powders were pressed under 250 MPa in a cold isostatic press machine (LDJ200/500–380YS). Finally, the ingots were held at 550 °C for 1 h in a vacuum resistance furnace with a vacuum degree of about 10^−3^ Pa.

A binary Mg–8Al alloy was prepared as the matrix alloy, and the other three composites reinforced with 8.2% of AlN particles were fabricated, respectively, as shown in Table 1. The 8.2AlN_in_/Mg–8Al composite means that the AlN particles were totally in situ synthesized from the chemical reaction between Al and BN [12,20], and the chemical reactions and calculated Gibbs free energies (ΔG) [21] were supposed as follows:

Al + BN = AlN + [B] ΔG_550 °C_ = −53.3 kJ/mol (1)

Mg + [B] = MgB_2_ ΔG_550 °C_ = −83.8 kJ/mol (2)

Based on Equations (1) and (2), the content of the as-mixed powders was designed as Mg–13.4Al–5BN. After completing chemical reactions, the 8.2AlN_in_/Mg–8Al composite, with some MgB_2_ particles, can be achieved. Similarly, the 8.2AlN_(in+ex)_/Mg–8Al composite contains 2.5% of in situ AlN and 5.7% of ex situ AlN particles, which is sintered from Mg–9.6Al–1.5BN–5.7AlN_(ex)_. The 8.2AlN_ex_/Mg–8Al composite indicates that the AlN particles were totally ex situ introduced.

The samples were cut from the same position of the composites. Then they were ground by SiC metallographic sandpaper, followed by a polishing procedure using MgO turbid liquid (5%). Microstructure analysis was carried out using a SU–70 field emission scanning electron microscopy (SEM, Hitachi Ltd., Japan) operated at 15 kV. The FEI Tecnai G^2^F20 transmission electron microscope (TEM, FEI Ltd., USA) operated at 200 kV and equipped with energy dispersion spectroscopy was used to clearly identify the distribution of AlN particles. The densities were tested using Archimedean drainage method. The hardness of the composites was tested by a HBS–3000 digital Brinell hardness tester, with the loading force of 2452 N and dwell time of 60 s. Compressive tests were conducted on a CMT4204 electronic all-purpose test machine at a constant crosshead speed of 2 mm/min. Each reported value regarding hardness and compressive strength is an average of five measurements.

## 3. Results and Discussion

**Figure 1:**
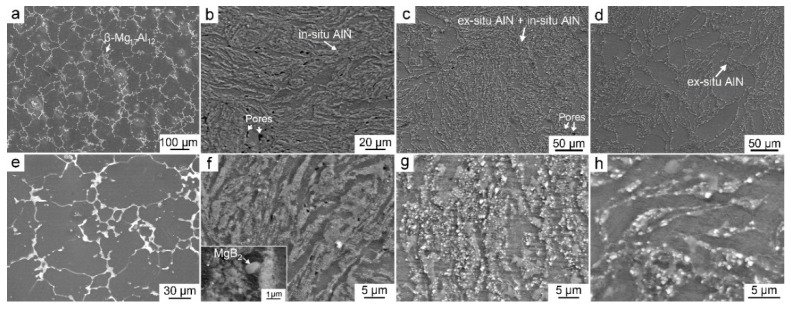
Microstructures of the Mg–8Al matrix alloy (**a**,**e**); 8.2AlN_in_/Mg–8Al (**b**,**f**); 8.2AlN_(in+ex)_/Mg–8Al (**c**,**g**) and 8.2AlN_ex_/Mg–8Al (**d**,**h**) composites.

**Figure 2:**
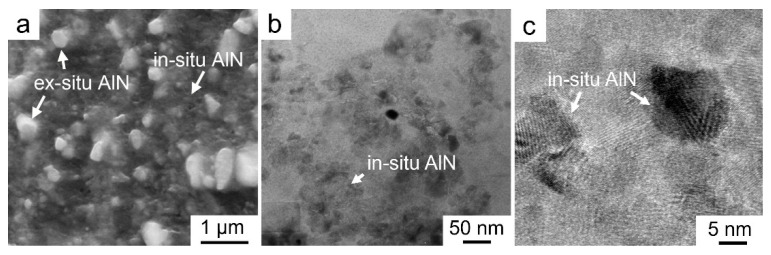
Microstructures of the 8.2AlN_(in+ex)_/Mg–8Al composite: (**a**) SEM image, showing the size of ex situ AlN particles; (**b**,**c**) TEM images, showing the size of in situ AlN particles.

Figure 1a,e shows microstructures of the Mg–8Al matrix alloy. Except for α–Mg grains, the formed intermetallic compound is β–Mg_17_Al_12_, exhibiting wormlike along the grain boundaries, as marked in Figure 1a. Figure 1b,f show the corresponding microstructures of the in situ AlN reinforced Mg composite, i.e., 8.2AlN_in_/Mg–8Al. It was seen that amounts of particles were fabricated, forming as streamlined shape. A certain amount of MgB_2_ particles (Figure 1f) were also been synthesized simultaneously, which will be further confirmed by TEM. Since the volume fraction of MgB_2_ is far less than AlN, therefore, they are not considered in the following part. Figure 1c,g shows the microstructures of the 8.2AlN_(in+ex)_/Mg–8Al composite, i.e., both in situ and ex situ AlN particles were applied as reinforcements. The distribution of particles also forms as a streamlined shape, resulting from the formation of in situ AlN particles, while some bright particles inserted in the other intermetallic compounds (Figure 1g) were also observed, which are the ex situ AlN particles. Figure 1d and h show the microstructures of the 8.2AlN_ex_/Mg–8Al composite. When all of the AlN particles were ex situ introduced, they combine with the β–Mg_17_Al_12_ and the formed streamlined morphology is inconspicuous. In order to clearly indicate the size of both in situ and ex situ AlN particles, magnified SEM and TEM images of the 8.2AlN_(in+ex)_/Mg–8Al composite were shown in Figure 2. It was found that most of the AlN_ex_ and AlN_in_ particles have the size of 0.72 ± 0.16 μm (Figure 2a) and 13 ± 1.5 nm (Figure 2b,c), respectively.

The streamlined microstructure of these AlN/Mg–Al composites is the typical behavior of PMMCs, which is composed of a particle-rich zone and a particle-poor zone [22,23]. The formation mechanism is related to the powder mixing process, as well as the relative low sintering temperature (550 °C in this paper). However, the in situ and ex situ AlN particles were found to be distributed uniformly inside the particle-rich zones, which can be confirmed by Figure 2a. In addition, it needs to be pointed out that some micron pores were detected specifically in the composites with in situ AlN particles (as marked in Figure 1b,c), which may be related to the in situ reaction process for synthesizing AlN. Based on the statistic measurements of pores by SEM images, the volume fractions of porosity in these composites were all estimated to be less than 2 vol.%.

The density of the three composites was tested and the values are listed in Table 1. Except for the Mg–8Al alloy, the 8.2AlN_in_/Mg–8Al composite has the highest density among these composites. This indicates that the composite reinforced with total in situ AlN but not ex situ particles can achieve the highest compactness. Furthermore, the hardness of the composites was tested, and the average values are presented in Table 1. The 8.2AlN_in_/Mg–8Al composite was also found to exhibit the highest hardness, while the 8.2AlN_(in+ex)_/Mg–8Al composite takes second place and 8.2AlN_ex_/Mg–8Al has the lowest hardness among these composites. Compared with the Mg–8Al matrix alloy, the in situ AlN particles lead to an increase of 54% in hardness, indicating the efficient strengthening performance of AlN particles in the 8.2AlN_in_/Mg–8Al composite.

**Figure 3:**
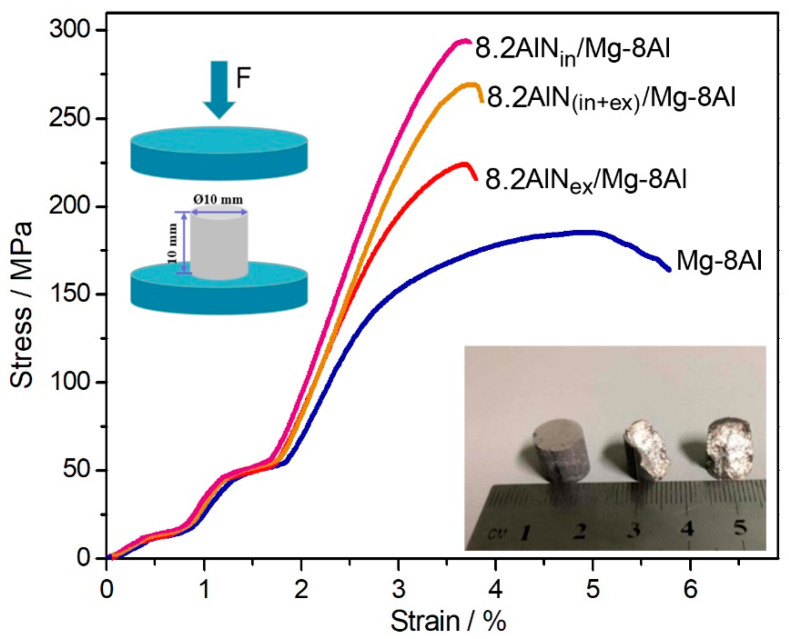
Typical compressive curves of the four alloys, and the inserted image shows the experimental details.

As mentioned above, some micron pores still exist in the prepared composites which are quite sensitive to the tensile test procedure. Therefore, to evaluate the mechanical properties, the compressive strength of the four alloys was measured. The typical compressive curves are shown in Figure 3, while statistic values were calculated (Table 1). It was found that the ultimate compressive strength of the composites exhibits a similar law with hardness values, i.e., the 8.2AlN_in_/Mg–8Al composite has the highest value, which can be as high as 295 ± 5 MPa.

**Figure 4:**
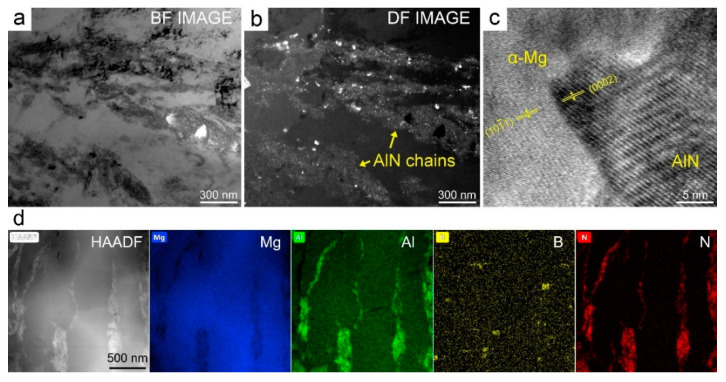
TEM analysis of the 8.2AlN_in_/Mg–8Al composite: (**a**) bright field image; (**b**) dark field image; (**c**) highly magnified picture and lattice image of AlN; (**d**) mapping of AlN chains.

The experimental results above clearly prove that the mechanical properties of Mg–Al alloy can be increased to the maximum extent by using in situ but not ex situ AlN particles as reinforcement. To clearly observe the strengthening behavior of the in situ AlN particles, TEM analysis was carried out on the 8.2AlN_in_/Mg–8Al composite after the compressive test, and the obtained results are shown in Figure 4. From the bright filed (BF) image and dark field (DF) image (Figure 4a,b), it was found that the AlN particles prefer to combine with each other, exhibiting particle chains. Figure 4c indicates that the in situ-formed AlN particles have clear interfaces with the Mg matrix. The distribution of element N, B, Mg and Al in a typical area (Figure 4d) also proves the identification of nano-AlN particle chains, inserted by a small number of MgB_2_ particles.

The strengthening performance of either in situ or ex situ AlN particles on Mg–8Al matrix alloy can be attributed to several mechanisms, e.g., grain boundary strengthening, dislocation strengthening and load bearing mechanism, as can be seen in other PMMCs [23]. In particular, the 8.2AlN_in_/Mg–8Al composite has additional advantages over the 8.2AlN_(in+ex)_/Mg–8Al and 8.2AlN_ex_/Mg–8Al composites, due to the special distribution of its AlN particles (Figure 4). The well-known Orowan strengthening is one of the most important strengthening mechanisms, which can be expressed as [24]:

(3) $$\Delta_{OR}=M\frac{0.4Gb}{\pi \lambda }\frac{\ln\left(\frac{2r}{b}\right)}{\sqrt{1-\nu }}$$

where Δ*_OR_* is the Orowan strength, *r* is the particle radius, *ν* is the Poisson ration, *M* is the orientation factor, *G* is the shear modulus and *b* is the Burgers vector of matrix, while *λ* is the interparticle spacing of strengthening phase. Therefore, for the 8.2AlN_in_/Mg–8Al composite, the interparticle spacing of AlN particles in the particle chains corresponds to a quite small value of *λ*. As a result, the Orowan strength will be obviously enhanced compared to the other two composites based on Equation (1).

**Figure 5:**
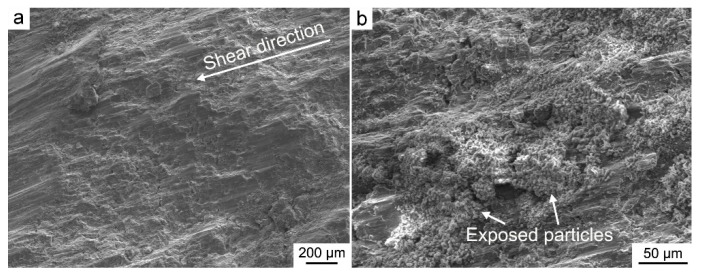
Shear fractures of the 8.2AlN_in_/Mg–8Al composite: (**a**) low magnification; (**b**) high magnification.

In fact, the statement of advantages for reinforcements with special distribution can be traced back to the year 1963, by Hashin and Shtrikman [25]. Based on Ref. [25], it was deduced that when the soft matrix (the Mg–Al matrix in this paper) is surrounded by the hard second phase (the AlN particles in this paper), the composite can exhibit the highest strength. This theory has also been confirmed in other Al and Ti composites [20,26]. Therefore, the distribution of nano-AlN as particle chains, surrounding the soft Mg–8Al matrix, is beneficial for the strengthening performance. This is because the particle chains may bear much higher stress or pile up more dislocations during the loading procedure. Figure 5 shows the corresponding shear fracture microstructure of the 8.2AlN_in_/Mg–8Al composite. The shear direction is visible, as marked in Figure 5a. In addition, the particle chains were observed as exposed at the shear surfaces (Figure 5b), which indicates that the final breakdown occurs, crossing the particle chains. In other words, the particle chains may act as barriers for the movement of dislocations and cracks during the shearing procedure.

In summary, this work has clarified that nano–AlN particles can be in situ synthesized in AlN/Mg–Al composites. The particles exhibit attractive strengthening performance on the Mg–Al matrix, and the in situ-formed AlN particles are much more efficient than ex situ ones. In addition, compared with the Mg matrix PMMCs strengthened by transition metal elements or rare earth elements [27,28], the 8.2AlN_in_/Mg–8Al composite has lower density and cost. Furthermore, compared with the Mg–Al alloy reinforced by SiC, TiC, TiB_2_ and AlN particles [8,9,10,15,16,17], the 8.2AlN_in_/Mg–8Al composite is not only easy to synthesize but also has better mechanical properties. Therefore, this paper may bring new insights for synthesizing Mg composites utilizing various methods. It is also expected that with further reasonable design of particle variety, content and distribution, new Mg composites may be developed.

## 4. Conclusions

To assess the strengthening performance of in situ and ex situ AlN particles on Mg–Al alloy, three 8.2AlN/Mg–8Al composites were fabricated in this paper. By comparing the microstructures and mechanical properties, it was found that the composite reinforced with total in situ AlN is better than those with total ex situ and mixture of in situ and ex situ ones. The distribution of in situ AlN as particle chains is responsible for the attractive properties.

## Figures and Tables

**Table 1:** Density, hardness and compressive strength of the synthesized samples.

Alloys	Density (g/cm^3^)	Hardness (HBW)	Compressive Strength (MPa)
Mg–8Al	1.747	58.1 ± 2.1	181 ± 3
8.2AlN_in_/Mg–8Al	1.746	89.5 ±2.3	295 ± 5
8.2AlN_(in+ex)_/Mg–8Al	1.743	80.3 ± 3.8	280 ± 7
8.2AlN_ex_/Mg–8Al	1.734	70.6 ± 1.5	225 ± 4

## Data Availability

The data presented in this study is available on request from the corresponding author. The data is not publicly available due to the research is ongoing.
